# MRI Texture Analysis for the Prediction of Stereotactic Radiosurgery Outcomes in Brain Metastases from Lung Cancer

**DOI:** 10.3390/jcm10020237

**Published:** 2021-01-11

**Authors:** Jung Hyun Park, Byung Se Choi, Jung Ho Han, Chae-Yong Kim, Jungheum Cho, Yun Jung Bae, Leonard Sunwoo, Jae Hyoung Kim

**Affiliations:** 1 Department of Radiology, Ajou University School of Medicine, Ajou University Medical Center, Suwon 443-380, Korea; nadine16jhp@gmail.com; 2 Department of Radiology, Seoul National University Bundang Hospital, 82, Gumi-ro 173beon-gil, Bundang-gu, Seongnam 13620, Korea; jojoini05@gmail.com (J.C.); bae729@gmail.com (Y.J.B.); leonard.sunwoo@gmail.com (L.S.); jaehkim@snu.ac.kr (J.H.K.); 3 Department of Neurosurgery, Seoul National University Bundang Hospital, 82, Gumi-ro 173beon-gil, Bundang-gu, Seongnam 13620, Korea; nstaus29@daum.net (J.H.H.); chaeyong@snu.ac.kr (C.-Y.K.)

**Keywords:** stereotactic radiosurgery, brain metastasis, magnetic resonance imaging, texture analysis

## Abstract

This study aims to evaluate the utility of texture analysis in predicting the outcome of stereotactic radiosurgery (SRS) for brain metastases from lung cancer. From 83 patients with lung cancer who underwent SRS for brain metastasis, a total of 118 metastatic lesions were included. Two neuroradiologists independently performed magnetic resonance imaging (MRI)-based texture analysis using the Imaging Biomarker Explorer software. Inter-reader reliability as well as univariable and multivariable analyses were performed for texture features and clinical parameters to determine independent predictors for local progression-free survival (PFS) and overall survival (OS). Furthermore, Harrell’s concordance index (C-index) was used to assess the performance of the independent texture features. The primary tumor histology of small cell lung cancer (SCLC) was the only clinical parameter significantly associated with local PFS in multivariable analysis. Run-length non-uniformity (RLN) and short-run emphasis were the independent texture features associated with local PFS. In the non-SCLC (NSCLC) subgroup analysis, RLN and local range mean were associated with local PFS. The C-index of independent texture features was 0.79 for the all-patients group and 0.73 for the NSCLC subgroup. In conclusion, texture analysis on pre-treatment MRI of lung cancer patients with brain metastases may have a role in predicting SRS response.

## 1. Introduction

Lung cancer is the most common cancer that metastasizes to the brain [1]. Lung cancer incidence is on the rise; however, survival rates are also increasing because of early diagnosis and the development of effective therapies [2]. As long-term survival outcomes in lung cancer patients continue to improve, the burden of brain metastases will inevitably grow, necessitating the need for optimal therapeutic options with low toxicity to manage brain metastases [3]. Stereotactic radiosurgery (SRS) is a focal treatment technique for brain metastases performed with a linear accelerator and involves delivering high-dose radiation to well-defined targets [4]. Increasing evidence suggests that there is no difference in the overall survival rates of affected patients receiving SRS coupled with whole-brain radiotherapy (WBRT) and those receiving SRS alone. Additionally, patients receiving SRS with WBRT had a higher risk of decline in learning and memory function [5,6]. Moreover, SRS has been reportedly useful in treating large metastatic lesions [7] as well as metastatic lesions around important brain structures [8]. As a result, its applications are becoming increasingly popular for avoiding the potential neurocognitive sequelae of WBRT. Magnetic resonance imaging (MRI) is important for both the early diagnosis of, as well as guiding optimal treatment strategies for brain metastasis. Therefore, utilizing information obtained from brain MRIs is essential for the successful treatment of patients with brain metastasis.

Many functional and microstructural MRI techniques using perfusion MRI and diffusion-weighted MRI (DWI) have been applied to brain metastases [9]. Dynamic susceptibility contrast (DSC)-MRI has shown its capability to differentiate radiation necrosis and tumor recurrence by measuring the relative cerebral blood volume (rCBV) and relative cerebral blood flow (rCBF) in patients with cerebral metastases treated with SRS [10]. Dynamic contrast enhanced (DCE)-MRI has shown its potential value in predicting the response of brain metastases to antineoplastic therapy in patients with lung cancer [11]. Furthermore, the apparent diffusion coefficient (ADC) value calculated from DWI has also proved to be useful in predicting SRS response [12]. However, these techniques need further validation to be used in clinical decision-making and the biological heterogeneity of metastases require more individualized image biomarker to accurately predict treatment response.

Radiomics is expected to serve as a bridge between medical imaging and personalized medicine [13] and specifically, is expected to be utilized increasingly in the field of oncology by applying the radiomics approach of texture analysis. Texture analysis is a technique that can effectively provide quantitative information regarding spatial variation of gray-level distribution and inter-relationship of voxels in a clinical image [14], which cannot be perceived by the naked eye. In studies associated with the diagnosis, prognosis, and treatment response of cancer, texture analysis metrics have reportedly been used to assess intratumoral heterogeneity [15,16]. This analysis has also been employed in the classification of brain metastases by their primary site of origin [17], differentiating brain metastases among various pathological types of lung cancer [18], and in predicting the treatment response to non-small cell lung cancer (NSCLC) [19,20,21].

By using non-invasive imaging techniques, texture analysis can be applied to gain information regarding tumor microenvironment and radiosensitivity, eventually aiding in the safe management of brain metastatic lesions. No previous study has combined texture analysis, clinical parameters, and morphological MRI features to develop a regression model that predicts clinical outcomes of SRS in brain metastasis. Therefore, we aimed to investigate the potential role of MRI-based texture analysis in a multivariable predictive model of survival for patients with lung cancer brain metastasis.

## 2. Materials and Methods

### 2.1. Patients

**Figure 1:**
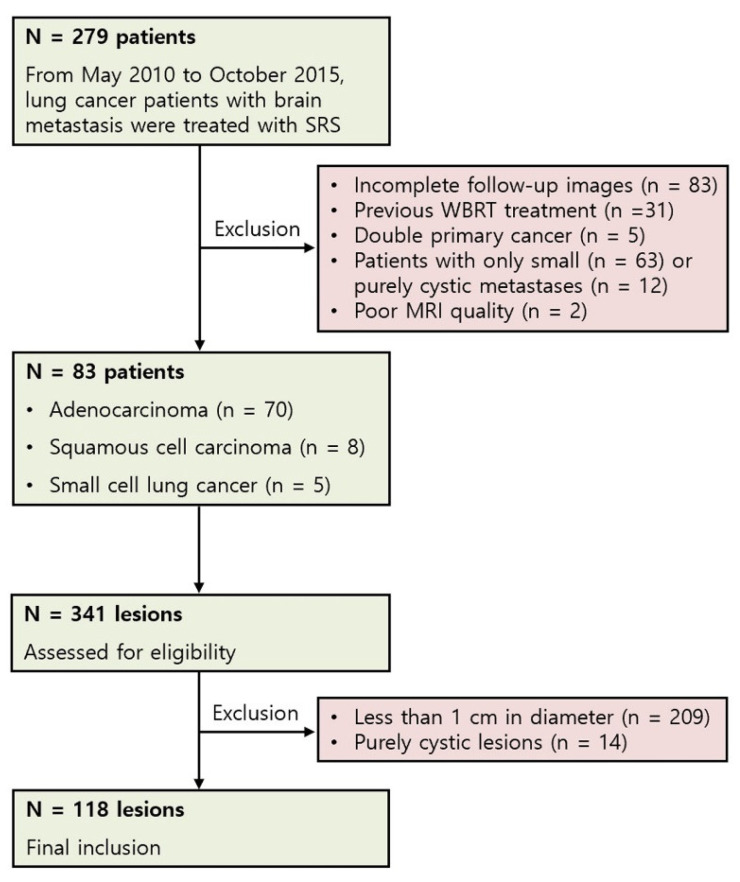
Patient and lesion selection flowchart (SRS, streotactic radiosurgery; WBRT, whole-brain radiotherapy; MRI, magnetic resonance imaging).

This retrospective study was approved by the Institutions Review Board and the requirement for informed consent was waived. Between May 2010 and October 2015, a total of 279 patients with brain metastases were treated with radiosurgery at our institution. Among them, those who met the following criteria were included in this study: (a) initial diagnosis of primary lung cancer by histopathology and (b) available pre-treatment brain MRI, including 1 mm thickness T1-weighted image (T1WI, pre- and post-contrast administration) and T2-weighted image (T2WI). Patients with the following criteria were excluded: (a) incomplete follow-up images within 6 months, (b) previous WBRT treatment, (c) diagnosis of double primary cancer, (d) small metastatic lesions with a maximum tumor diameter less than 1 cm, (e) purely cystic brain metastases, and (f) inadequate MRI quality. This was a lesion-based study and multiple metastatic lesions with more than 1 cm in diameter in a single patient were all included. This size criterion was chosen to satisfy the minimum reported number of pixels required for successful texture analysis. Purely cystic brain metastases were excluded because only metabolically active regions of the tumor were of interest for texture analysis [22]. The summary of inclusion and exclusion processes are shown in Figure 1. Clinical data were collected via an electronic medical record and the following factors were evaluated: age, gender, primary lung cancer histology, recursive partitioning analysis class [23], presence of extracranial metastasis, use of target therapy (tyrosine kinase inhibitors, such as erlotinib, gefitinib, afatinib, and osimertinib), local failure within the brain, SRS dose, and overall survival (OS) after SRS. No patient had surgery for brain metastasis before or after the SRS.

### 2.2. MR Imaging Protocol

All pre-treatment MR images were acquired with a 1.5 T instrument (Gyroscan Intera; Philips Healthcare, Best, The Netherlands). The imaging parameters for 3-dimensional (3D) T1WI were: repetition time/echo time (TR/TE), 20/4.6 ms; flip angle, 30°; acquisition matrix, 256 × 256; slice thickness, 1 mm; field-of-view, 256 × 256 mm^2^; voxel size, 1 × 1 × 1 mm^3^; and no slice gap. The post-contrast T1WI was acquired after intravenous administration of a single-dose of Gadobutrol (0.1 mmol/kg, Gadovist, BAYER; Leverkusen, Germany) with a 6-min delay. The axial T2WI was obtained with the following parameters: TR/TE, 11,788.49/120 ms; flip angle, 90°; acquisition matrix, 256 × 256; slice thickness, 1 mm; field-of-view, 256 × 256 mm^2^; voxel size, 1 × 1 × 1 mm^3^; and no slice gap.

### 2.3. Image Analysis

#### 2.3.1. Tumor Identification, Characterization, and Region-of-Interest (ROI) Allocation

**Figure 2:**
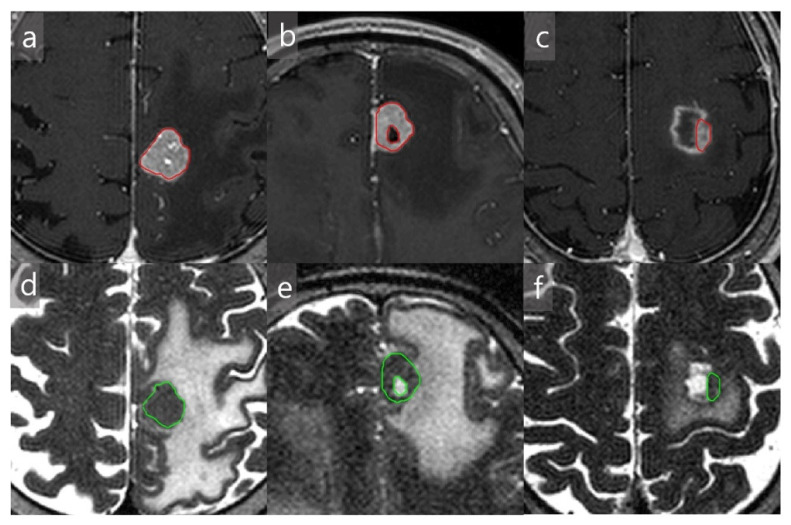
Examples of tumor segmentation on solid (**a**,**d**), predominantly solid (**b**,**e**), and predominantly cystic (**c**,**f**) metastases. The regions-of-interests are drawn on contrast-enhanced T1-weighted (**a**–**c**) and T2-weighted (**d**–**f**) images for each lesion.

Two board-certified neuroradiologists (J.H.P and B.S.C. with 5 and 20 years of experience, respectively) independently reviewed all MR images. They identified the tumors on T2WI and contrast-enhanced (CE) T1WI, and free-hand polygonal ROIs were allocated on a single section of both sequences. The readers drew ROIs in an independent fashion, attempting to include the largest cross-sectional area of the solid tumor portion. Cystic, hemorrhagic, or necrotic areas were excluded as only metabolically active regions of the tumor were of interest for the analysis [22]. Representative examples of tumor segmentation are shown in Figure 2. The readers also classified the tumors as solid, predominantly solid (cystic portion < 50%), and predominantly cystic (cystic portion ≥ 50%) based on the morphology. For more complex cases, a consensus was always reached.

#### 2.3.2. Evaluation of the Treatment Response and Study Endpoints

The treatment response after SRS was assessed on MR images according to the Response Assessment in Neuro-Oncology Brain Metastases criteria [24] by the two neuroradiologists. Disagreements about tumor size changes were ultimately resolved by a consensus. Target lesions were classified as progressive or non-progressive disease (including stable disease, partial response, and complete response). The study endpoints were local progression-free survival (PFS), defined as the time from the beginning of the SRS to the time of progression of each target lesion, and OS. Patient follow-up and evaluation of treatment outcomes were performed retrospectively by reviewing medical records and MRIs.

### 2.4. Texture Analysis

Texture analysis was performed using the Imaging Biomarker Explorer (IBEX) software [25]. T2WI and CE T1WI of all subjects were exported to IBEX. Two readers segmented the tumor borders on both sequences, as mentioned previously. Normalization of the gray levels was performed within the software by rescaling all image signal intensities to fit between μ ± 3σ (μ: gray-level mean, σ: gray-level standard deviation). Additional normalization to account for the number of voxels was performed for the five volume-dependent features (busyness, coarseness, gray-level non-uniformity, run-length non-uniformity (RLN), and energy) [26].

For each ROI, 156 texture features from four categories were computed (Supplementary Table S1). First-order texture features were intensity direct/histograms (74 features) and second-order features were derived from gray-level co-occurrence matrices (66 features), gray-level run-length matrices (11 features), and neighborhood gray-tone difference matrices (5 features). Gray-level co-occurrence matrix-based features were computed and analyzed separately using distances of 1 (d1), 4 (d4), and 7 (d7) pixels. The neighboring properties of pixels in the four directions (0°, 45°, 90°, and 135°) of the 2-dimensional (2D) space were averaged equally.

### 2.5. Radiosurgery

A treatment plan was generated using the Leksell GammaPlan (Elekta Instrument) system based on the findings from the thin-sliced MRI. Radiosurgery was performed using the Leksell Gamma Knife PERFEXION (Elekta Instrument AB, Stockholm, Sweden). The radiosurgery isodose and marginal dose prescribed were initially determined using the Radiation Therapy Oncology Group (RTOG) 90-05 dosing guidelines [27] and calculated during dose planning using the best-fit isodose method. The marginal dose was then optimized by reducing approximately 10–20% of the recommended doses, according to the individual patient history of previous radiotherapy and/or tumor size, to reduce the radiation-related side effects. The treatments were usually designed to deliver 50% of the maximum dose to the target margins in a single fraction. The final prescribed dose, expressed as a marginal dose and the associated treatment parameters, are summarized in Table 1.

### 2.6. Statistical Analysis

All statistical analyses were performed using R v.3.5.2. (R Foundation for Statistical Computing, Vienna, Austria) and SAS statistical package (9.4) (Cary, NC, USA). Interclass correlation coefficient (ICC) values were calculated for each texture feature. Features with an ICC value ≥ 0.8 were considered reproducible and selected. All the clinical parameters and reproducible texture features were tested using a univariable Cox proportional-hazards model to identify the predictors of PFS and OS. Clinical parameters with a *p*-value lower than 0.05 were used for further analyses. A stepwise selection method and the least absolute shrinkage and selection operator (LASSO) method was used to select core texture features. Multivariable Cox proportional-hazards analyses were performed for significant clinical parameters and core texture features to identify independent predictors of PFS and OS. Harrell’s concordance index (C-index) was used to assess the discriminative power of the identified independent texture features [28]. Internal validation was carried out with 1000 bootstrap replications. All Cox proportional-hazards models considered marginal models regarding multiple lesions within the same patient [29]. In all statistical tests, a *p*-value < 0.05 was considered statistically significant.

## 3. Results

### 3.1. Patient Clinical Characteristics and Survival

From a total of 83 patients, 118 metastatic tumors (primary tumor histology: 103 adenocarcinoma, 9 squamous cell carcinoma, 6 small cell lung cancer (SCLC)) were identified to be suitable for study and were included in the analysis. The main characteristics of the patient and tumors are summarized in Table 1. Of the 118 tumors, 34 (28.8%) showed local progression during the follow-up period and the mean local PFS was 18 months (range: 3–120 months). Twenty-seven patients (32.5%) died before the follow-up, and the mean OS was 26.8 months (range: 6–121 months).

### 3.2. Univariable Cox Proportional-Hazards Regression Model for Clinical and Texture Parameters

The univariable Cox regression model was applied to each clinical parameter and is described in Table 2. The variables with *p*-values < 0.05 included tumor characteristic (solid, predominantly solid, predominantly cystic), primary tumor histology, target therapy use, maximal tumor diameter, and SRS prescription dose.

An ICC value was calculated independently by the two readers for each of the 156 texture features on T2WI and CE T1WI sequences. The mean ICC value was 0.72 (range, 0.34–0.91) on CE T1WI and 0.71 (range, 0.19–0.9) on T2WI. In total, 137 features assessed in CE T1WI and 138 features assessed in T2WI had good inter-reader agreement with an ICC value of 0.6 or above. Features with an ICC value ≥ 0.8 were considered robust and selected for the univariable analysis. Results of the univariable analysis of texture features for the prediction of local PFS and OS are presented in Supplementary Table S2.

### 3.3. Multivariable Cox Proportional-Hazards Regression Model for Clinical and Texture Parameters

The multivariable analysis was performed to identify independent predictors of local PFS and OS. All the significant clinical parameters from the univariable analysis and core texture features selected from the stepwise selection and LASSO methods were included in the multivariable analysis (Table 3).

Among the clinical parameters and texture features included in the multivariable analysis for local PFS prediction, SCLC histology, RLN, and short-run emphasis (SRE) were significantly associated with PFS (hazard ratio (HR) = 4.15, *p* = 0.038; HR = 1.16, *p* < 0.001; HR = 0.92, *p* = 0.047, respectively). For OS prediction, only SCLC histology proved to be the independent clinical parameter (HR = 0, *p* < 0.001); however, this result is not reliable, as all SCLC patient data were censored. None of the texture features were predictive of OS (Supplementary Table S3).

A subgroup analysis on the NSCLC group, including adenocarcinoma and squamous cell carcinoma patients, revealed RLN (HR = 1.15, *p* = 0.014) and local range mean (HR = 1.15, *p* = 0.019) to be significant texture features associated with PFS (Table 4). SRE showed marginal statistical significance (HR = 0.99, *p* = 0.05). None of the clinical and texture parameters were predictive of OS (Supplementary Table S3).

A multivariable image biomarkers model was developed based on the two significant independent texture features in the all-patients group (RLN and SRE) and the NSCLC subgroup (RLN and local range mean). Each image biomarkers model resulted in a C-index of 0.79 (95% CI: 0.72–0.86) and 0.73 (95% CI: 0.63–0.83) in the all-patients and NSCLC groups, respectively, in the original dataset. The internally validated C-index of 1000 bootstrap samples for each model was 0.78 (95% CI: 0.71–0.87) in the all-patients group and 0.74 (95% CI: 0.63–0.84) in the NSCLC group.

## 4. Discussion

In this study, we demonstrated that texture features extracted from pre-treatment MRI have the potential to predict local control of brain metastasis in lung cancer patients after SRS treatment. A multivariable predictive model developed based on these texture features performed reasonably well in the all-patients group and the NSCLC subgroup, respectively. Our study results are meaningful because we only included patients treated with SRS with no previous WBRT record. Most previously published reports of survival prediction after SRS for brain metastases included patients who were treated with other modalities, such as WBRT, surgery, or a combination of SRS and WBRT [30]. Therefore, our findings provide more useful information for understanding local tumor control after treatment with SRS alone. Moreover, this study was the first to consider clinical parameters and morphological MRI features by using a regression model to evaluate the usefulness of texture analysis in predicting the clinical outcome of SRS.

Higher RLN values were predictive of poor local tumor control when clinical factors (target therapy use, primary tumor histology, maximal tumor diameter, and marginal dose) and morphological MRI features (tumor characteristic) were considered in the regression model. This result is in concordance with a previous study by Zhai et al. that showed higher RLN values were associated with poor survival in the nasopharyngeal and head and neck cancer datasets [31]. Additionally, higher SRE values were associated with a lower risk of local tumor progression. RLN and SRE are both second-order statistics derived from run-length matrices, which characterize large areas within the tumor (groups of voxels) to provide information about regional heterogeneity [32,33]. A run is defined as a length of consecutive pixels presenting with the same gray-level intensity in a specific direction, and the relationships between the run lengths make up the texture [34]. RLN measures the similarity among run lengths; high RLN values indicate dissimilar run lengths within the ROI. SRE measures the distribution of short runs in the image; high SRE values are related to fine texture, which includes many short runs of similar gray-level intensities, whereas low SRE is related to coarse texture [34]. Thus, dissimilar run lengths and a low number of short runs in the texture analysis may reflect intratumoral heterogeneity. In general, tumor heterogeneity at the microscopic level is one of the major causes of treatment failure in cancer; this holds especially true for glioblastoma multiforme [35,36]. We speculated that RLN and SRE values extracted from the pre-treatment MRI scans may provide valuable information regarding the underlying tumor heterogeneity, radiosensitivity, and/or vascularization, which could, in turn, be related to SRS treatment response.

In the NSCLC subgroup, the RLN value and the local range mean were significant texture features associated with a higher risk of local tumor progression. Local range mean is a first-order statistic that is the computed mean of the range value in each voxel’s neighborhood region [37]. Thus, a higher local range mean value is related to a wide range of gray-scale values within the ROI, which, in turn, may also be related to intratumoral heterogeneity. However, SRE showed only marginal statistical significance in predicting local tumor control in the subgroup analysis. It is unclear why the predictive value of SRE was not valid in the NSCLC subgroup. This may be partly due to the small sample size. The difference in the composition of histopathological tumor types may also have played a role, as texture features convey information about the underlying tumor pathology [18].

Another important finding in our study was that texture features extracted from CE T1WI were more valuable than those extracted from T2WI. This result is consistent with a previous study that differentiated between radiation necrosis and tumor progression using MRI-based radiomic features [38]. In our study, more features with an ICC value ≥ 0.8 were reliable when measured in CE T1WI, but not in T2WI. Moreover, most of the core texture features selected from the stepwise selection and LASSO methods were features calculated from the CE T1WI. Texture features extracted from the CE T1WI may convey information regarding the underlying tumor vascularity and may better reflect intratumoral heterogeneity with various gray-scale values than those extracted from the T2WI [18], thereby providing more valuable information in predicting SRS treatment response.

In our multivariable analysis, the SCLC primary tumor histology was the only clinical parameter significantly associated with local PFS. This result is consistent with that of a previous report by Kuremsky et al. [39] They reported a slightly higher HR of 6.46 compared to ours. The pathophysiology underlying poor SRS outcomes in SCLC is not fully understood, but a population of radioresistant clonogenic cells, increased invasion into brain parenchyma by diffuse infiltrative growth patterns, and large infiltration depth [40,41] have been proposed as the underlying mechanisms. Treatment failure in SCLC after chemoradiotherapy is also known to be substantial, suggesting that SCLC cells can develop radioresistance after an initially good response [42].

Treatment of brain metastases with chemotherapeutic drugs is known to be limited by the blood brain barrier, with its response rate reported to be 15–30% [43]. However, an increasing number of molecular-targeted drug therapies have been used to treat brain metastases in lung cancer patients and showed increased intracranial response rates, depending on the molecular profile and drug generations. It has been reported that lung cancer patients with brain metastasis who received erlotinib or gefitinib combined with radiotherapy or chemotherapy showed significantly increased intracranial response rates compared with those who received either drug alone [44,45]. Another previous study showed that patients who received SRS with target therapy showed improved overall survival and intracranial outcomes [46]. In our study, the effect of molecular target therapy on SRS response was evaluated in the univariable and multivariable analyses. Target therapy use was associated with improved local PFS in the univariable analysis but was not an independent predictor in the multivariable analysis. This result could be partly due to small sample size and further investigation is warranted. Prospective data regarding SRS combined with targeted therapy in patients with brain metastases are currently limited in the literature and may be an excellent topic for future trials.

The morphological MRI characteristics of the cystic composition of the tumor were investigated in the multivariable analysis in combination with other clinical and texture parameters. The results revealed that cystic composition was negatively associated with local PFS in the univariable analysis, but statistical significance was not reached in the multivariable analysis. Cystic brain metastasis was generally considered an unfavorable factor in achieving local tumor control after SRS. However, a study by Ebinu et al. proposed that cystic composition of a metastatic lesion does not predict the response to SRS [47]. They included cystic metastases that did not require cyst aspiration and concluded that no percentage of cystic volume predicted SRS response rates. We excluded purely cystic lesions in our study and achieved similar results, suggesting that the extent of cystic composition in metastatic lesions does not significantly impact their response to SRS.

Our study had several limitations. First, this was a retrospective study with a potential risk of selection bias. However, all patients who met the inclusion criteria were included in the study to minimize selection bias. Second, a small sample size that only included primary lung cancer data limits our ability to generalize our results to brain metastatic lesions originating from other primary tumors. Moreover, only a small number of SCLC patients were included, after excluding patients who had received prior WBRT treatment. Thus, our results on the primary tumor histology of SCLC should be interpreted with caution. Third, this was a single-center study using identical protocols with the same MRI scanner. Fourth, external validation of the image biomarkers model presented in this study was not performed. Further research including external data sets with different MRI scanners and protocols in a larger population are warranted before our results can be applied to routine clinical practice. Lastly, texture features were extracted from a single time-point using a 2D segmentation method, chosen for its convenience in investigation and ease of application. Future studies with delta-radiomics features extracted from 3D volume datasets could broaden our understanding of tumor heterogeneity and post-treatment changes.

## 5. Conclusions

In conclusion, we developed a prediction model using texture features extracted from MRI to predict local tumor control of SRS in patients with lung cancer brain metastases. We found that MRI texture analysis on pre-treatment CE T1WI may have a role in predicting local tumor control after SRS and this finding may aid decision-making regarding treatment planning and prognosis evaluation for patients treated with SRS for brain metastases.

## Figures and Tables

**Table 1:** Patient demographics and tumor characteristics.

	Value
**Age**	61 ± 11 (Range: 25–84)
**Sex**	
Male	47 (56.6%)
Female	36 (43.4%)
**Histology**	
Adenocarcinoma	70 (84.3%)
Squamous cell carcinoma	8 (9.7%)
Small cell lung cancer	5 (6%)
**RTOG RPA class**	
I	16 (19.3%)
II	67 (80.7%)
III	0
**Extracranial metastasis**	
Yes	35 (42.2%)
No	48 (57.8%)
**Targeted therapy use**	
Yes	32 (38.6)
No	51 (61.4)
**Morphologic tumor characteristic**	
Solid	51 (43.2%)
Predominantly solid	47 (39.8%)
Predominantly cystic	20 (17%)
**Maximal tumor diameter (mm)**	17.7 ± 8.2 (Range: 10–45)
**Marginal dose prescribed (Gy)**	18.8 ± 2.1 (Range: 12–24)
**Maximum dose (Gy)**	37.5 ± 4.7 (Range: 22.7–50.1)

RTOG, radiation therapy oncology group; RPA, recursive partitioning analysis. Data are mean ± standard deviation.

**Table 2:** Univariable Cox proportional hazards regression analysis of clinical parameters.

	Local Progression-Free Survival		Overall Survival	
	HR (95% CI)	*p* Value	HR (95% CI)	*p* Value
Age	1.02 (0.99–1.05)	0.095	0.97 (0.95–1.01)	0.107
Sex	0.47 (0.22–1.04)	0.062	0.92 (0.39–2.14)	0.837
Tumor characteristic	– *	0.035	– *	0.422
Targeted therapy	2.17 (1.01–4.66)	0.046	0.55 (0.24–1.26)	0.157
Histology	– *	<0.001	– *	<0.001
Extracranial metastasis	0.95 (0.42–2.14)	0.905	0.81 (0.34–1.91)	0.628
RTOG RPA class	1.12 (0.47–2.66)	0.804	0.712 (0.21–2.37)	0.579
Maximal tumor diameter	1.04 (1.01–1.08)	0.013	0.984 (0.95–1.02)	0.386
Marginal dose prescribed	0.85 (0.74–0.99)	0.031	1.06 (0.92–1.22)	0.417

HR, hazard ratio; CI, confidence interval; RTOG, radiation therapy oncology group; RPA, recursive partitioning analysis. * No global hazard ratio for variables with more than 2 modalities.

**Table 3:** Multivariable Cox proportional hazards regression analysis of factors affecting local progression-free survival in all patients.

	HR (95% CI)	*p* Value
**Histology**		
Adenocarcinoma	1.0 (Reference)	
Squamous cell carcinoma	1.82 (0.42–7.92)	0.423
Small cell lung cancer	4.15 (1.08–15.98)	0.038
**Target therapy use**		
Yes	1.0 (Reference)	
No	1.48 (0.52–4.17)	0.464
**Morphologic tumor characteristic**		
Solid	1.0 (Reference)	
Predominantly solid	0.39 (0.13–1.12)	0.081
Predominantly cystic	1.34 (0.43–4.19)	0.62
**Maximal tumor diameter**	1.01 (0.94–1.09)	0.764
**Marginal dose (Gy)**	0.90 (0.66–1.21)	0.472
**Texture features (CE T1W1)**		
Dissimilarity (d7)	1.0 (0.99–1.02) *	0.659
Inverse Difference Norm (d7)	1.42 (0.32–6.38) *	0.649
Run-Length Non-uniformity	1.16 (1.07–1.25) *	<0.001
Short-Run Emphasis	0.92 (0.84–0.99) *	0.048

HR, hazard ratio; CI, confidence interval; CE T1WI, contrast-enhanced T1-weighted image. * Hazard ratio per 10,000-unit increase.

**Table 4:** Multivariable Cox proportional hazards regression analysis of factors affecting local progression-free survival in the NSCLC subgroup.

	HR (95% CI)	*p* Value
**Histology**		
Adenocarcinoma	1.0 (Reference)	
Squamous cell carcinoma	1.11 (0.21–5.87)	0.906
**Target therapy use**		
Yes	1.0 (Reference)	
No	1.84 (0.69–4.85)	0.464
**Morphologic tumor characteristic**		
Solid	1.0 (Reference)	
Predominantly solid	0.48 (0.17–1.4)	0.181
Predominantly cystic	1.16 (0.31–4.37)	0.828
**Maximal tumor diameter**	1.03 (0.96–1.22)	0.548
**Marginal dose (Gy)**	0.92 (0.69–1.22)	0.548
**Texture features (CE T1WI)**		
Dissimilarity (d7)	1.0 (0.98–1.02) *	0.659
Inverse Difference Norm (d7)	1.09 (0.14–8.92) *	0.649
Run-Length Non-uniformity	1.15 (1.03–1.29) *	0.014
Short-Run Emphasis	0.99 (0.99–1) *	0.05
Local Range Mean	1.15 (1.02–1.29)	0.019

HR, hazard ratio; CI, confidence interval; CE T1WI, contrast-enhanced T1-weighted image. * Hazard ratio per 10,000-unit increase.

## Data Availability

Data sharing is not applicable to this article.

## Supplementary Materials

The following are available online at https://www.mdpi.com/2077-0383/10/2/237/s1. Table S1: Summary of texture features and their abbreviations. Table S2: Cox univariable statistical analysis results of texture features. Table S3: Multivariable analysis of factors affecting overall survival in all patients and NSCLC subgroup.
