# Supplementary material for: MRI Texture Analysis for the Prediction of Stereotactic Radiosurgery Outcomes in Brain Metastases from Lung Cancer
Source: J Clin Med. 2021 Jan 11;10(2):237. doi: 10.3390/jcm10020237 (PMC7827024; doi:10.3390/jcm10020237)
Supplement: Supplementary file 1 [file jcm-10-00237-s001.pdf]

Supplementary Table S1. Summary of texture features and their abbreviations.

Feature category	Feature (abbreviation)
**Intensity direct/histogram**	Energy
	Global Entropy
	Global Maximum
	Global Mean
	Global Median
	Global Minimum
	Global Standard Deviation
	Global Uniformity
	Inter-Quartile Range
	Kurtosis
	Local Entropy Maximum
	Local Entropy Mean
	Local Entropy Median
	Local Entropy Minimum
	Local Entropy Standard Deviation (Std)
	Local Range Maximum
	Local Range Mean
	Local Range Median
	Local Range Minimum
	Local Range Std
	Local Std Maximum
	Local Std Mean
	Local Std Median
	Local Std Minimum
	Local Std Std
	Mean Absolute Deviation
	Median Absolute Deviation
	Percentile (×19)
	Percentile Area (×19)
	Quantile (×5)
	Range
	Root Mean Square
	Skewness
	Variance
**GLCM**	Auto-Correlation
	Cluster Prominence
	Cluster Shade
	Cluster Tendency
	Contrast

	Correlation
	Difference Entropy
	Dissimilarity
	Energy
	Entropy
	Homogeneity
	Homogeneity 2
	Information Measure Correlation 1 (InfoMeasureCorr 1)
	Information Measure Correlation 2 (InfoMeasureCorr 2)
	Inverse Difference Moment Norm (InvDifMN)
	Inverse Difference Norm (InvDifN)
	Inverse Variance
	Maximum Probability (Max Probability)
	Sum Average
	Sum Entropy
	Sum Variance
	Variance
**GLRLM**	Gray Level Non-uniformity (GLN)
	High Gray Level Run Emphasis (HGRE)
	Long-Run Emphasis (LRE)
	Long-Run High Gray Level Emphasis (LRHGE)
	Long-Run Low Gray Level Emphasis (LRLGE)
	Low Gray Level Run Emphasis (LGRE)
	Run-Length Non-uniformity (RLN)
	Run Percentage (RP)
	Short-Run Emphasis (SRE)
	Short-Run High Gray Level Emphasis (SRHGE)
	Short-Run Low Gray Level Emphasis (SRLGE)
**NDM**	Busyness
	Coarseness
	Complexity
	Contrast
	Texture Strength

GLCM, gray-level co-occurrence matrix; GLRLM, gray-level run-length matrix; NDM, neighborhood gray-tone difference matrix

Supplementary Table S2. Cox univariable statistical analysis results of texture features

Variables	Local progression-free survival			Overall survival		
	P Value	HR	95% CI	*P* Value	HR	95% CI
Contrast (d1)	.017	0.188	0.048–0.743	.007	2.961	1.338–6.55
Contrast (d7)	.026	0.711	0.525–0.961	.02	1.183	1.026–1.364
Difference Entropy (d1)	.005	0.056	0.007–0.423	.03	7.829	1.221–50.214
Difference Entropy (d7)	.011	0.031	0.002–0.459	.011	14.383	1.821–113.63
Dissimilarity (d1)	.025	0.239	0.068–0.841	.011	3.045	1.283–7.226
Dissimilarity (d7)	.011	0.24	0.081–0.716	.028	2.26	1.087–4.697
Homogeneity*	.012	1.099	1.021–1.183	.019	0.923	0.863–0.987
Homogeneity 2*	.011	1.089	1.019–1.163	.016	0.932	0.88–0.987
InvDifMN (d1)†	.017	5.313	1.347–20.962	.007	0.337	0.152–0.747
InvDifMN (d7)†	.026	1.409	1.041–1.906	.020	0.845	0.732–0.974
InvDifN (d1)†	.011	1.036	1.008–1.906	.011	0.973	0.953–0.994
InvDifN (d4)†	.025	1.015	1.002–1.028	.012	0.988	0.98–0.997
InvDifN (d7)†	.01	1.015	1.004–1.026	.029	0.992	0.984–0.999
Run-Length Non-uniformity†	.89	0.995	0.931–1.064	.143	1.033	0.989–1.079
Run Percentage	.021	0.006	0.001–0.468	.021	140.96	2.064–9627.812
Short-Run Emphasis	<.001	0.001	0.001–0.041	.045	122.507	1.11–13522.11
Energy†	.274	0.82	0.574–1.171	.465	0.912	0.711–1.169
Inter Quartile Range	.026	0.954	0.915–0.995	.013	1.032	1.006–1.059
Local Entropy Mean	.009	0.241	0.082–0.71	.052	3.126	0.987–9.907
Local Entropy Median	.005	0.251	0.095–0.664	.047	2.913	1.011–8.393
Local Entropy Minimum	.218	0.59	0.255–1.366	.079	1.864	0.929–3.738
Local Range Mean	.009	0.969	0.946–0.992	.025	1.026	1.003–1.049
Local Range Median	.011	0.969	0.946–0.993	.011	1.023	1.005–1.042
Local Std Mean	.005	0.892	0.823–0.968	.027	1.087	1.01–1.17
Local Std Median	.007	0.893	0.822–0.97	.012	1.083	1.017–1.152
Median Absolute Deviation	.024	0.909	0.837–0.988	.013	1.069	1.014–1.127
InfoMeasureCorr 1 (d7)‡	.423	0.057	0.002–63.623	.35	0.105	0.001–11.952
LRHGE‡	.309	1.001	0.999–1.002	.151	0.998	0.995–1.001
Run Percentage‡	.053	0.081	0.006–1.037	.002	36.586	3.744–357.515
Short-Run Emphasis‡	.041	0.072	0.006–0.898	.002	55.16	4.631–657.06
Energy†‡	.815	0.954	0.642–1.417	.463	1.115	0.833–1.494
Local Range Mean‡	.045	0.985	0.97–1	<.001	1.015	1.007–1.024

Local Std Mean[‡]	.053	0.947	0.896–1.001	<.001	1.056	1.025–1.088
Coarseness[†‡]	.208	1.034	0.981–1.091	.263	0.966	0.909–1.026

HR, Hazard ratio; CI, Confidence interval; InvDifMN, Inverse Difference Moment Norm; InvDifN, Inverse Difference Norm; Std, Standard deviation; [‡]Values measured on T2WI, otherwise measured on contrast-enhanced T1WI; InfoMeasureCorr 1, Information Measure Correlation 1; LRHGE, Long Run High Gray Level Emphasis

*Hazard ratio per 100-unit increase; [†]Hazard ratio per 10000-unit increase

Supplementary Table S3. Multivariable analysis of factors affecting overall survival in all patients and NSCLC subgroup

	HR (95% CI)	*P* Value
**Overall survival in all patients**		
**Histology**		
Adenocarcinoma	1.0 (Reference)	
Squamous cell carcinoma	1.92 (0.79–4.67)	.149
Small cell lung cancer	0 (0–0)	<.001
**Extracranial metastasis**		
Yes	1.0 (Reference)	
No	0.72 (0.29–1.82)	.49
**Texture features (CE T1WI)**		
Contrast (d1)	1.25 (0.89–1.76)*	.193
Inverse Difference Moment Norm (d1)	9.54 (0.32–286.32)†	.194
Inter-Quartile Range	1.03 (0.99–1.07)	.074
**Texture features (T2WI)**		
Short-Run Emphasis	1.04 (0.97–1.11)	.31
Local Standard Deviation Mean	1.01 (0.92–1.09)	.924
**Overall survival in NSCLC group**		
**Histology**		
Adenocarcinoma	1.0 (Reference)	
Squamous cell carcinoma	1.98 (0.87–4.49)	.102
**Extracranial metastasis**		
Yes	1.0 (Reference)	
No	0.75 (0.3–1.88)	.541
**Texture features (CE T1WI)**		
Contrast (d1)	1.24 (0.89–1.69)*	.194
Inverse Difference Moment Norm (d1)	8.28 (0.34–201.07)†	.194
Inter-Quartile Range	1.03 (0.99–1.07)	.106
**Texture features (T2WI)**		
Local Range Mean	0.94 (0.79–1.1)	.419
Local Standard Deviation Mean	1.34 (0.75–2.39)	.329

HR, Hazard ratio; CI, Confidence interval; CE T1WI, contrast-enhanced T1-weighted image

*Hazard ratio per 10000-unit increase; †Hazard ratio per 10000000-unit increase
